# Reboxetine-induced urinary hesitancy

**DOI:** 10.4103/0019-5545.55964

**Published:** 2005

**Authors:** Suresh Borade, Mahesh Bhirud, Kranti Kadam

**Affiliations:** * Senior Resident, Department of Psychiatry, Lokmanya Tilak Municipal Medical College and Lokmanya Tilak Municipal General Hospital, Sion, Mumbai 400022; ** Senior Resident, Department of Psychiatry, Lokmanya Tilak Municipal Medical College and Lokmanya Tilak Municipal General Hospital, Sion, Mumbai 400022; *** Lecturer, Department of Psychiatry, Lokmanya Tilak Municipal Medical College and Lokmanya Tilak Municipal General Hospital, Sion, Mumbai 400022

Sir,

One of the troublesome side-effects of tricyclic anti-depressants is urinary hesitancy due to their anticholinergic effects.^1^

Reboxetine, the only selective noradrenaline reuptake inhibitor, is an effective and well-tolerated antidepressant. Since it has low affinity for muscarinic, cholinergic receptors,^2^ anticholinergic side-effects such as urinary retention or hesitancy are not expected.

However, some patients on reboxetine (2–4 mg/day) complain of urinary hesitancy. A peripheral noradrenergic mechanism termed ‘pseudoanticholinergic syndrome’ may be responsible for this side-effect.^3^

Tamsulosin, an alpha-1A receptor antagonist, is recommended for reboxetine-induced urinary hesitancy.^4^,^5^ Therefore, the following precautions should be taken while a patient is on reboxetine:

1. Enquire about any history of urinary complaints beforehand.
2. In elderly patients, titrate the dose upwards gradually.
3. During follow-up visits, ask patients whether they have any urinary hesitancy.
4. If reboxetine-induced urinary hesitancy does set in, then rather than withdrawing reboxetine, a trial of capsule tamsulosin (available locally as Urinat) can be started in a single dose of 0.4 mg half-an-hour after dinner every day.
